# Automated pupillometry for detection of delirium in surgical intensive care patients

**DOI:** 10.3389/fmed.2026.1822468

**Published:** 2026-06-04

**Authors:** Patrik Mica, Marek Lukes, Andrea Pokorna, Jan Hruda, Lyle Olson, Michal Svoboda, Ivan Cundrle

**Affiliations:** 1 Department of Anesthesiology and Intensive Care, St. Anne’s University Hospital Brno, Brno, Czechia; 2 Faculty of Medicine, Masaryk University, Brno, Czechia; 3 Department of Health Sciences, Faculty of Medicine, Masaryk University, Brno, Czechia; 4 Centre of Cardiovascular Surgery and Transplantation, Brno, Czechia; 5 Department of Cardiovascular Disorders, Mayo Clinic, Rochester, MN, United States; 6 Institute of Biostatistics and Analyses, Ltd., Brno, Czechia

**Keywords:** automated pupillometry, delirium, intensive care, pain, sedation

## Abstract

**Background:**

Delirium is a frequent and serious complication in critically ill patients and associated with adverse outcomes. Automated pupillometry has emerged as a potential diagnostic tool for the detection of delirium. However, pupillometric parameters are also influenced by pain, potentially confounding their relationship to delirium. The aim of this study was to evaluate the association between automated pupillometry parameters and delirium in patients with high risk of pain (surgical patients) admitted to intensive care units (ICU).

**Methods:**

This prospective multicenter observational study included adult surgical patients admitted to an ICU between April 2023 and August 2025. Delirium was assessed every 6 h after admission using CAM-ICU. Automated pupillometry together with pain intensity scores (VAS, CPOT, BPS) were performed concurrent with CAM-ICU. To account for repeated measurements within individual patients, a generalized linear mixed-effects model (GLMM) with patient as a random effect was used to evaluate the association between pupillometric parameters and diagnosed delirium.

**Results:**

Forty-nine patients were included, yielding 417 measurements, of which 48 (12%) were done in patients who were simultaneously CAM-ICU positive. Among pupillometric variables, only average pupillary latency (LAT avg) was significantly shorter in patients with delirium (*p* = 0.01). In the mixed-effects model adjusted for sedation depth, level of consciousness, and pain intensity, LAT avg. was independently associated with delirium (OR 1.55 per 0.01; 95% CI 1.13–2.13; *p* = 0.007).

**Conclusion:**

In surgical ICU patients, shortened pupillary light reflex latency is associated with delirium. Automated pupillometry may serve as a useful tool for detection of delirium in this population.

**Clinical trial registration:**

https://clinicaltrials.gov/study/ Identifier: NCT05811208.

## Introduction

Delirium is an acute disturbance of consciousness with a fluctuating course and cognitive dysfunction, representing the most common form of brain dysfunction in critically ill patients (1). It occurs in up to 50% of patients receiving mechanical ventilation and is associated with prolonged intensive care unit (ICU) stay and increased mortality (2). Despite high prevalence, delirium often remains unrecognized, particularly in its hypoactive form (3, 4). Therefore, regular screening using validated tools such as the Confusion Assessment Method for the ICU (CAM-ICU) is recommended (2). Although CAM-ICU is considered the gold standard for delirium assessment, its use may be limited in sedated, ventilated, or neurologically impaired patients, where reliable evaluation can be challenging (5, 6).

The pupillary light reflex is a measure of the function of the autonomic nervous system, which is involved in the pathophysiology of delirium (7). Pupillometric assessment is a quantitative tool for the evaluation of acute brain dysfunction in the context of delirium (8). Indeed, previous studies have suggested an association between several pupillometric parameters and delirium (9, 10). However, similar changes of pupillometry parameters were also observed in patients with pain placing the role of pupillometry in delirium monitoring in patients with high risk of pain (e.g., surgical patients) into question (11).

We hypothesized that automated pupillometry parameters can detect delirium irrespective of pain intensity scores. Accordingly, the aim of this study was to compare pupillometry parameters and pain intensity scores in surgical patients admitted to the ICU with and without delirium.

## Methods

### Study design

This was a prospective multicenter observational study conducted to evaluate the relationship between automated pupillometry parameters and the occurrence of delirium in surgical ICU patients.

The study was carried out in the intensive care units of St. Anne’s University Hospital in Brno and the Center for Cardiovascular and Transplantation Surgery in Brno between April 2023 and August 2025. The study was conducted in accordance with the Declaration of Helsinki. All patients provided standard written informed consent for the surgical procedure prior to surgery. Written informed consent for participation in the study was not obtained prior to surgery but was obtained at the time of ICU admission. In cases where patients were unable to provide consent, it was obtained from a legally authorized representative. The study protocol was approved by the Ethics Committee of St. Anne’s University Hospital in Brno (11V/2023). The study was registered at clinicaltrials.gov (NCT05811208).

### Patients

Inclusion criteria were surgical patients, admitted to the ICU with age ≥18 years. Patients with eye pathology (current or in medical history), a history of traumatic brain injury, stroke, epilepsy, or neuromuscular disorders were excluded. Ophthalmic exclusion criteria were assessed based on medical history obtained from the patient or their relatives; no formal ophthalmological examination was performed.

### Delirium screening

Screening for delirium was performed at regular 6-h intervals (beginning with the admission to the ICU) using the CAM-ICU, a validated instrument for detecting delirium in critically ill patients (12). Delirium was defined as at least one positive CAM-ICU assessment during the observation period. CAM-ICU was performed only when the patient’s clinical condition allowed for valid evaluation, specifically when the level of sedation as assessed by the Richmond Agitation-Sedation Scale (RASS) was not lower than −2.

### Automatic pupillometry

Each patient underwent repeated pupillometric measurements concurrent with CAM-ICU. Quantitative pupillometry was performed using the NPi®-200 Pupillometer (NeurOptics Inc., Irvine, CA, United States). The manufacturers’ recommended parameters were recorded during each measurement as follows; pupil size (size, mm)—the initial maximal pupil diameter before light stimulation; minimum pupil diameter (MIN, mm)—the smallest diameter reached during the constriction phase in response to the light stimulus; relative change in pupil diameter (%CH, %)—the percentage change in size as a reaction for light stimulation; constriction velocity (CV, mm/s)—the average speed of pupil constriction; maximum constriction velocity (MCV, mm/s)—the peak velocity reached during the constriction response; latency (LAT, s)—time interval between the onset of the light stimulus and the beginning of constriction; dilation velocity (DV, mm/s)—average speed of pupil redilation following the constriction phase (13). All measurements were conducted under standardized lighting conditions. Pupillometric parameters are presented as an average of both eyes; right and left eye data are provided separately in Supplementary Table 1. All measurements were performed by trained investigators.

### Clinical parameters

Vital signs (heart rate, blood pressure, respiratory rate, and peripheral oxygen saturation), concurrent medication (analgesics, sedatives and catecholamines) and pain intensity were recorded simultaneously with pupillometry and CAM-ICU measurements. For pain intensity, the Visual Analogue Scale (VAS), Behavioral Pain Scale (BPS), and Critical Care Pain Observation Tool (CPOT) were used (14–17). Additionally, demographic data (age, sex, comorbidities, main diagnosis) and ICU length of stay were also collected.

### Statistics

The Shapiro–Wilk test was used to evaluate normality. Accordingly, Student *t*-test, Mann–Whitney *U* test and two-tailed Fisher exact test were used for group comparison. To account for repeated measurements, a generalized linear mixed-effects model (GLMM) with patient as a random effect was applied to identify parameters associated with delirium. Decision statistics (2 × 2) were done to estimate sensitivity, specificity, positive and negative predictive values, and positive and negative likelihood ratios calculated for several cutoff values of LAT avg. Data were summarized as mean ± SD or median (IQR); *p*-values <0.05 were considered statistically significant. Statistica software 12.0 (StatSoft Inc., Prague, Czechia) was used for the analysis.

## Results

A total of 49 subjects were enrolled in the study. Eight patients developed delirium yielding 48 measurements whereas 41 patients remained delirium-free yielding 369 measurements. The basic subject characteristics including comorbidities and type of surgery are shown in Table 1. Vascular and cardiac surgery were the most common types of surgery. The most common comorbidities were arterial hypertension, diabetes, and ischemic heart disease.

**Table 1:** Subject characteristics.

Characteristic	Value
Age, years, median (IQR)	64 (60–71)
Sex, *n* (%)	Male 39 (79.6)
	Female 10 (20.4)
Length of hospital stay, days, median (IQR)	4 (3–9)
Postoperative period, days, median (IQR)	3 (2–7)
Duration of measurement, days, median (IQR)	3 (2–4)
In-hospital mortality during study period, *n* (%)	4 (8.2)
Type of surgery, *n* (%)	
Cardiac surgery	12 (24.5)
Vascular surgery	14 (28.6)
Abdominal surgery	11 (22.4)
Thoracic surgery	4 (8.2)
Other	7 (14.3)
Not specified	1 (2.0)
Comorbidities, *n* (%)	
Arterial hypertension	34 (69.4)
Diabetes mellitus	14 (28.6)
CAD	13 (26.5)
Atrial fibrillation	11 (22.4)
COPD	9 (18.4)
Dyslipidemia	8 (16.3)
PAD	7 (14.3)
Arrhythmia	7 (14.3)
CKD	6 (12.2)
AKI	5 (10.2)
BPH	4 (8.2)
Peptic ulcer disease	3 (6.1)

Data are presented as median (IQR). *n* refers to the number of individual measurements. CAD, coronary artery disease; COPD, chronic obstructive pulmonary disease; PAD, peripheral artery disease; CKD, chronic kidney disease; AKI, acute kidney injury; BPH, benign prostatic hyperplasia.

Automatic pupillometry results are shown in Table 2. There were no significant between group differences in most of the measured parameters. Only the latency of the pupillary response (LAT avg) differed significantly (*p* = 0.01), shorter in patients with delirium compared to patients without delirium. This difference was observed in the average value of both eyes as well as in both the right and left pupil (Supplementary Table 1).

**Table 2:** Comparison of pupillometric parameters according to CAM-ICU status.

Parameter	CAM-ICU positive (*n*)	Median (IQR)	CAM-ICU negative (*n*)	Median (IQR)	*p*-value
SIZE avg. (mm)	48	3.10 (2.70–3.52)	369	3.17 (2.63–3.87)	0.78
MIN avg. (mm)	48	2.11 (1.93–2.64)	369	2.26 (1.93–2.61)	0.48
%CH avg. (%)	48	29.75 (21.50–35.75)	369	28.50 (22–34)	0.76
CV avg. (mm/s)	48	1.78 (1.18–2.20)	367	1.61 (1.18–2.20)	0.68
MCV avg. (mm/s)	48	2.99 (1.89–3.87)	368	2.62 (1.97–3.49)	0.37
LAT avg. (s)	47	0.23 (0.22–0.27)	369	0.25 (0.23–0.29)	0.01
DV avg. (mm/s)	44	0.79 (0.55–1.07)	319	0.85 (0.62–1.02)	0.93

Data are presented as median (IQR). *n* refers to the number of individual measurements. SIZE avg, average pupil size; MIN avg, average minimum pupil diameter; %CH avg, average percentage of constriction; CV avg, average constriction velocity of both pupils; MCV avg, average maximum constriction velocity; LAT avg, average reaction latency; DV avg, average dilation velocity.

The results of vital signs and pain assessment are shown in Table 3. Between groups, there were small but significant differences in RASS, CPOT and HR which were higher and GCS which was significantly lower in patients with delirium. There was no difference in catecholamine support which was present in 20 measurements (42%) in patients with delirium and in 200 measurements (54%) in patients without delirium (*p* = 0.12).

**Table 3:** Comparison of clinical and physiological parameters between CAM-ICU groups.

Parameter	CAM-ICU positive (*n*)	Median (IQR)	CAM-ICU negative (*n*)	Median (IQR)	*p*-value
GCS	48	14 (13-14)	369	15 (14-15)	<0.01
RASS	47	1 (0-1)	368	0 (0-0)	<0.01
VAS	47	0 (0-0)	364	0 (0-1)	0.08
CPOT	48	0 (0–3)	369	0 (0-1)	0.02
BPS	48	3 (3–5)	369	3 (3-4)	0.07
HR (beats/min)	48	88.5 (75–102.5)	368	82 (74–95)	0.02
SBP (mmHg)	48	118.5 (100–137.5)	369	123 (113–136)	0.07
DBP (mmHg)	48	63 (55–71)	369	61 (56–70)	0.81
MAP (mmHg)	46	63.0 (55–71)	360	80 (73–88)	0.76
SpO_2_ (%)	48	96.0 (95–97)	367	96 (94–98)	0.80
RR (breaths/min)	48	21.0 (18–25)	369	20 (16–24)	0.01

Data are presented as median (IQR). *n* refers to the number of individual measurements. GCS, Glasgow Coma Scale; RASS, Richmond Agitation Sedation Scale; VAS, Visual Analogue Scale; CPOT, Critical Care Pain Observation Tool; BPS, Behavioral Pain Scale; HR, heart rate; SBP, systolic blood pressure; DBP, diastolic blood pressure; MAP, mean arterial pressure; SpO_2_, peripheral oxygen saturation; RR, respiratory rate.

Pupillometry parameter LAT avg., together with relevant confounders observed to differ between groups (RASS, GCS, and CPOT), were included in a generalized linear mixed-effects model with patient as a random effect (Table 4). LAT avg. and RASS were independently associated with delirium, whereas GCS and CPOT were not. Decision statistics performed for several LAT cut-off values are shown in Table 5, with low LAT avg. values highly specific for delirium.

**Table 4:** Generalized linear mixed-effects model (GLMM).

Variable	OR (95% CI)	*p*-value
LAT avg. per 0.01	1.55 (1.13–2.13)	0.007
RASS	0.15 (0.05–0.46)	<0.001
GCS	0.98 (0.26–3.70)	0.97
CPOT	0.65 (0.37–1.12)	0.12

LAT avg, average reaction latency; RASS, Richmond Agitation Sedation Scale; GCS, Glasgow Coma Scale; CPOT, Critical Care Pain Observation Tool.

**Table 5:** LAT cut-off values.

LAT avg	Sensitivity % (95% CI)	Specificity % (95% CI)	LR+	LR−	PPV % (95% CI)	NPV % (95% CI)	Accuracy % (95% CI)
≤0.20	19.15 (9.15–33.26)	89.97 (86.44–92.84)	1.91 (0.98–3.70)	0.90 (0.78–1.04)	19.57 (11.15–32.05)	89.73 (88.33–90.98)	81.97 (77.93–85.55)
≤0.25	70.21 (55.11–82.66)	42.01 (36.92–47.22)	1.21 (0.99–1.49)	0.71 (0.45–1.12)	13.36 (11.16–15.92)	91.72 (87.54–94.58)	45.19 (40.34–50.11)
≤0.30	97.87 (88.71–99.95)	7.05 (4.65–10.15)	1.05 (1.00–1.11)	0.30 (0.04–2.17)	11.83 (11.31–12.36)	96.30 (78.31–99.47)	17.31 (13.80–21.29)

LAT, latency; CI, confidence interval; LR, likehood ratio; PPV, positive predictive value; NPV, negative predictive value.

## Discussion

The major finding of this study was that the LAT avg. was significantly shorter in surgical ICU patients with delirium than in those without. Moreover, the mixed-effects model showed that LAT avg. and RASS were independently associated with delirium, whereas GCS and CPOT were not. The specificity of low LAT avg. values was high, suggesting a low number of false positive cases. This finding suggests automated pupillometry might be a useful tool for delirium detection in surgical ICU patients.

Of pupillometric parameters, only low LAT avg. was significantly associated with delirium. Delirium is considered a result of interactions of multiple pathophysiological mechanisms, including inflammatory processes, neurotransmitter imbalance, and dysregulation of the autonomic nervous system (18). Increased sympathetic activity combined with impaired cholinergic modulation may contribute to altered sensory processing and increased pupillary reactivity (19, 20). These mechanisms may provide a plausible framework for interpreting the shorter pupillary latency observed in this study.

Previous studies examining the relationship between automated pupillometry parameters and delirium have reported inconsistent findings. Some studies have reported reduced pupillary light reflex amplitude and slower constriction velocity in patients with delirium (8, 9). Others identified percent constriction and dilation velocity as the most predictive parameters (10). This discrepancy may be related to different patient populations, techniques used and additional factors such as pain, stress response, and concurrent administration of opioids or sedatives (21–23).

Pain represents an important modulating factor in the interpretation of pupillometric parameters, as nociceptive stimulation directly affects pupillary dynamics through autonomic activation (24, 25). Previous studies have shown that automated pupillometry can detect nociceptive responses and identify insufficient analgesia even in patients unable to communicate (26). At the same time, pupillary responses to pain may be influenced by the level of analgesia, especially by opioids (27). Importantly, in our study, the mixed-effects model showed that the relationship between LAT avg. and delirium was independent of pain intensity (as measured by CPOT), suggesting that the observed alterations in pupillary latency were not predominantly influenced by nociceptive stimuli.

Sedation significantly influences pupillary reactivity and may contribute to changes in certain pupillometric parameters. Previous studies have demonstrated that increasing depth of sedation is associated with attenuation of the pupillary light reflex and alterations in pupillary dynamics (28). In our study, however, the association between LAT avg. and delirium was independent of the level of sedation, suggesting that the observed changes in pupillary latency are not primarily attributable to sedative effects.

Apart from pupillary latency, no significant differences were observed between patients with and without delirium in other automated pupillometry parameters. This finding may be related to the effects of pain and sedative–analgesic medication commonly administered in the postoperative period, which are known to influence pupil size and reactivity and may therefore mask potential differences between groups (23, 29). These factors may have a greater impact on parameters considered more sensitive to pharmacological and external influences, such as constriction amplitude or velocity, whereas pupillary latency may have been affected less.

Our study may have clinical implications. Automated pupillometry could potentially represent a useful tool for delirium detection in clinical settings where standard assessment is limited, such as in sedated, mechanically ventilated, or poorly communicative patients (23, 30). In postoperative surgical patients, particularly those at risk of significant pain, pupillary latency may provide clinically relevant information, as its association with delirium in our cohort appeared to be independent of pain intensity. The high specificity of pupillary latency implies a low rate of false-positive findings, suggesting that a positive result may reduce the need for additional confirmatory testing.

This study has several limitations. First, a relatively low number of subjects were included (*n* = 49) with a small number of patients who developed delirium (*n* = 8). Second, only surgical patients were included, which limits the generalizability of our findings. Third, ophthalmic conditions were assessed based on medical history only, and no formal ophthalmological examination was performed; therefore, undiagnosed conditions potentially affecting pupillary function cannot be excluded. Fourth, the study population was heterogeneous in terms of sex distribution and types of surgical procedures, which may have influenced the observed outcomes. Fifth, all measurements were done in the ICU, where it was not possible to fully standardize external conditions such as ambient light. Sixth, pupillometric measurements were performed by multiple investigators, although the measurements are automatic and all investigators were trained, this may have introduced some interobserver variability influencing interrater reliability. Seventh, the computational algorithms for pupillometry may differ from those employed in other studies, potentially affecting the absolute values of individual parameters and limiting direct comparability. This applies particularly to the NPi, which was not included in the analysis, as its calculation is based on a proprietary algorithm that is not publicly available and is primarily designed for neurological prognostication (31).

## Conclusion

In this study, surgical patients admitted to the ICU who experienced delirium exhibited shorter LAT avg. values on pupillometry compared with those without delirium. The association between LAT avg. and delirium was independent of sedation, level of consciousness and pain intensity. This observation suggests automated pupillometry has the potential to detect delirium in surgical patients.

## Data Availability

The raw data supporting the conclusions of this article will be made available by the authors, without undue reservation.
