# Supplementary material for: Automated pupillometry for detection of delirium in surgical intensive care patients
Source: Front Med (Lausanne). 2026 Jun 4;13:1822468. doi: 10.3389/fmed.2026.1822468 (PMC13275463; doi:10.3389/fmed.2026.1822468)
Supplement: Supplementary file 1 [file Table_1.DOCX]

**Suplementary data**

| Table S1. Right and left eye pupillometric parameters according to CAM-ICU status | | | | | |
| --- | --- | --- | --- | --- | --- |
| Parameter | CAM-ICU  positive (n) | Median (IQR) | CAM-ICU negative (n) | Median (IQR) | p-value |
| P-SIZE (mm) | 48 | 2.99 (2.58–3.66) | 369 | 3.18 (2.60–3.96) | 0.53 |
| L-SIZE (mm) | 48 | 3.09 (2.63–3.86) | 369 | 3.11 (2.59–3.81) | 0.81 |
| P-MIN (mm) | 48 | 2.11 (1.84–2.55) | 369 | 2.23 (1.94–2.64) | 0.20 |
| L-MIN (mm) | 48 | 2.15 (1.91–3.02) | 369 | 2.27 (1.94–2.57) | 0.86 |
| P-%CH (%) | 48 | 29.50 (23–35) | 369 | 29.00 (23–35) | 0.64 |
| L-%CH (%) | 48 | 29.00 (19.50–35.00) | 369 | 28.00 (22–34) | 0.91 |
| P-CV (mm/s) | 48 | 1.82 (1.21–2.27) | 367 | 1.62 (1.18–2.16) | 0.56 |
| L-CV (mm/s) | 48 | 1.64 (1.10–2.26) | 368 | 1.61 (1.11–2.18) | 0.93 |
| P-MCV (mm/s) | 48 | 3.03 (1.94–3.83) | 368 | 2.58 (1.86–3.57) | 0.34 |
| L-MCV (mm/s) | 48 | 2.69 (1.90–3.93) | 368 | 2.61 (1.83–3.49) | 0.49 |
| P-LAT (s) | 48 | 0.23 (0.20–0.27) | 369 | 0.27 (0.23–0.27) | 0.01 |
| L-LAT (s) | 47 | 0.23 (0.23–0.27) | 369 | 0.25 (0.23–0.27) | 0.03 |
| P-DV (mm/s) | 47 | 0.77 (0.51–1.02) | 340 | 0.79 (0.58–1.02) | 0.68 |
| L-DV (mm/s) | 45 | 0.86 (0.53–1.14) | 337 | 0.84 (0.62–1.03) | 0.93 |

Data are presented as median (IQR). **n** refers to the number of individual measurements.

P-SIZE = diameter of the right pupil; L-SIZE = diameter of the left pupil; P-MIN = minimum diameter of the right pupil; L-MIN = minimum diameter of the left pupil; P-%CH = percentage of constriction of the right pupil; L-%CH= percentage of constriction of the left pupil; P-CV = average constriction velocity of the right pupil; L-CV = average constriction velocity of the left pupil; P-MCV = maximum constriction velocity of the right pupil; L-MCV = maximum constriction velocity of the left pupil; P-LAT= latency of the right pupil reaction; L-LAT= latency of the left pupil reaction; P-DV = dilation velocity of the right pupil; L-DV = dilation velocity of the left pupil
